# Death of Drs. Stipp and Cleaveland

**Published:** 1853-01

**Authors:** G. L. Paine


" To live in hearts we leave behind *is not to die.*"

It rarely falls to our lot to record the death of so estimable a man as Dr. J. A. Cleveland, who died of the prevailing epidemic in Charleston, S. C. on the 28th of September last.

Dr. Cleveland was known throughout the South, and particularly in Georgia and South Carolina, where he had resided the most of his life, as one of the most celebrated practitioners of Dentistry in that section, and wherever known was esteemed and beloved for his many virtues and excellent qualities. He was what we rarely meet with in this life, almost a perfect man. in the various relatious of life he had no superior. As a son, brother, husband and friend he was all that man could be on earth. Faithful to every trust and honest to a fault, his deportment endeared him to all, and none knew him but to love him, his death has left a void in society which can never be filled.

Xenia, O., Dec. 24th, 1852.

Dr. Jas. Taylor—Dear Sir.—It becomes my melancholy duty to inform you of the death of Dr. N. B. Stipp, my associate in the practice of Dentistry for nearly three years. He died, Dec. 2d, of typhoid fever, after a severe illness of some thirteen days duration. He was very delirious during his whole sickness, insomuch that his family, or his friends could have but little conversation with him of a profitable nature. He has left a disconsolate widow with five small children, and a large circle of warm friends to mourn their irreparable loss. He had been engaged *actively*, in the practice of dentistry about 4 years: and although his pupilage with his very competent and gentlemanly preceptor, A. S. Talbert, D. D. S., of Dayton, O., was of short duration, yet, by his indomitable energy, and great mechanical cast of mind, united to his insinuating address, gentlemanly deportment, and real personal merit, he succeeded in establishing for himself a professional reputation of which any one might well be proud. He possessed a mind of more than ordinary capabilities, with great aptitude in its applications to the pursuits of life—to all the chief moral reforms of the day, to the promotion of which he was ardently devoted. He was especially devoted to his profession, which was "the child of his love." In his death, the Dental Profession has lost an energetic and devoted practitioner,—the M. E. Church to which he belonged, a worthy member,—and our community an excellent citizen.

G. L. PAINE, D. D. S.

